# Characterization of pre-existing anti-PEG and anti-AGAL antibodies towards PRX-102 in patients with Fabry disease

**DOI:** 10.3389/fimmu.2023.1266082

**Published:** 2023-09-22

**Authors:** Malte Lenders, Lina Marleen Feidicker, Stefan-Martin Brand, Eva Brand

**Affiliations:** ^1^ Department of Internal Medicine D, and Interdisciplinary Fabry Center (IFAZ), University Hospital Muenster, Muenster, Germany; ^2^ Institute of Sports Medicine, Interdisciplinary Fabry Center (IFAZ), University Hospital Muenster, Muenster, Germany

**Keywords:** anti-drug antibodies, COVID-19, enzyme replacement therapy, half-life, humoral response, inhibition

## Abstract

Polyethylene glycol (PEG)ylated drugs are used for medical treatment, since PEGylation either decreases drug clearance or/and shields the protein from undesirable immunogenicity. PEGylation was implemented in a new enzyme replacement therapy for Fabry disease (FD), pegunigalsidase-alfa (PRX-102). However, exposure to PEG via life-style products and vaccination can result in the formation of anti-PEG antibodies. We demonstrate the *de novo* formation of functional anti-PEG antibodies in a healthy male after the second mRNA-based vaccination against SARS-CoV-2. Consequently, we analyzed the frequency and inhibitory function of anti-PEG and anti-α-Galactosidase A (AGAL) antibodies in 102 FD patients (46.9% males). We identified 29 out of 87 (33.3%) patients with low anti-PEG titers. Sera from patients without anti-AGAL antibodies [n=70] showed a higher rescued AGAL activity of agalsidase-beta and PRX-102 [both p<0.0001] compared to those with anti-AGAL antibodies [n=15]. Sera from anti-AGAL antibody-negative and -positive patients had less inhibitory effects on PRX-102 (rescued activity: 89 ± 6% versus 85 ± 7% and 49 ± 26% versus 25 ± 32%; both p<0.0001). Enzyme stability assays demonstrated that AUCs in anti-AGAL-negative sera (n=20) were 7.6-fold higher for PRX-102, while AUCs of both enzymes in anti-AGAL-positive sera (n=6) were decreased. However, AUC for PRX-102 was 33% of non-anti-AGAL-positive sera treated PRX-102 and 5-fold higher compared to agalsidase-beta. Anti-PEG antibodies had no significant effects on serum half-life of PRX-102, probably due to low titers. Conceivably, therapy efficacy may be superior under next-generation PRX-102 therapy compared to current enzyme replacement therapies in terms of reduced inhibitory effects of anti-AGAL and minor inhibitory effects of anti-PEG antibodies.

## 1 Introduction

Fabry disease (FD) is an X-linked lysosomal storage disease, caused by a deficiency of the enzyme α-galactosidase A (AGAL; EC 3.2.1.22). The enzyme deficiency results in a progressive accumulation of the AGAL substrate globotriaosylceramide (Gb3), leading to a multisystem disease including heart failure, cardiac arrhythmia, cerebrovascular events, and end-stage renal disease (1). Since 2001, FD is treatable by enzyme replacement therapy (ERT) [agalsidase-alfa (0.2 mg/kg body weight (b.w.) every other week (e.o.w.); Shire/Takeda) or agalsidase-beta (1.0 mg/kg b.w. e.o.w.; Sanofi-Genzyme)] intravenously (2, 3). Furthermore, since 2016 FD patients with an amenable mutation can also be treated orally with migalastat (migalastat; 123 mg hard capsules, every other day, Amicus Therapeutics), which is a small molecule and serves as a pharmaceutical chaperone, increasing endogenous residual AGAL activity (4). Although treatment with both compounds for ERT showed beneficial effects on disease manifestation and progression in affected patients, classical male patients without cross-reactive immunologic material (i.e. lack of any endogenous AGAL protein) are under a high risk to form neutralizing anti-drug antibodies (ADAs) against both compounds, which significantly impair the therapeutic efficacy of ERT (5–9).

To overcome this pitfall and to prolong plasma half-life, next generation ERTs such as pegunigalsidase-alfa were designed (PRX-102, Protalix BioTherapeutics, Chiesi Farmaceutici). PRX-102 is a PEGylated (PEG, polyethylene glycol) and covalently cross-linked form of recombinant AGAL produced in plant cells (tobacco) and developed as novel ERT for FD (10–12). The drug (Elfabrio, Chiesi) was approved by both the EMA and FDA in May 2023 for the treatment of adult Fabry patients (1.0 mg/kg body weight, intravenously every 2 weeks). Preliminary studies on PRX-102 suggested less immunogenicity compared to agalsidase-alfa and agalsidase-beta (12) and in this respect, we recently demonstrated that pre-existing anti-drug antibodies against agalsidase-alfa and agalsidase-beta showed about 30% less affinity (less inhibitory capacity) for PRX-102 (13).

**Figure 1:**
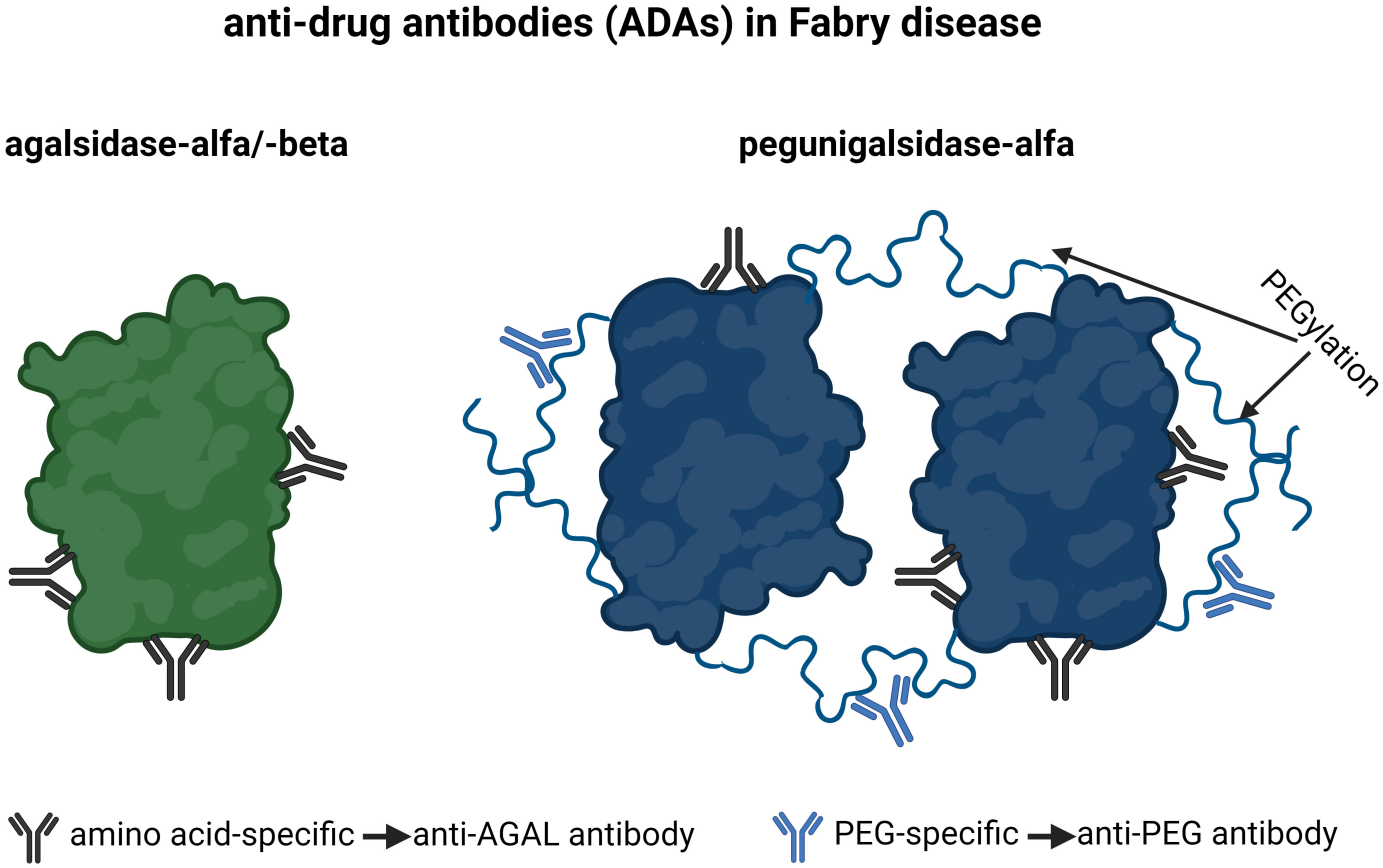
Current anti-drug antibodies in Fabry disease. Antibodies, which are amino-acid-specific recognize epitopes on agalsidase-alfa/-beta and pegunigalsidase-alfa and are termed anti-AGAL antibodies. Antibodies, which are PEG-specific, recognize PEG residues on pegunigalsidase-alfa and are termed anti-PEG antibodies. AGAL, α-galactosidase A; PEG, polyethylene glycol.

PEGylated drugs are used for human pharmaceutical treatment, since PEGylation either mediates decreased drug clearance or/and shields the protein from undesirable immunogenicity and anti-drug antibodies (14). The early exposure to PEG via cosmetics, soaps, and laxatives seems to be responsible for a “hidden” immunization, explaining the formation of PEG-specific antibodies in the general population (15). These antibodies may interfere with PEGylated drugs, reducing their therapeutic efficacy (by direct inhibition or due to increased clearance) or leading to a complement activation-related pseudoallergy (16). Since the biochemical modifications of next generation ERTs such as PRX-102 include PEGylation, anti-drug antibodies in FD can be separated in antibodies recognizing epitopes, which are amino acid-specific (anti-AGAL antibodies) and antibodies recognizing PEG residues (anti-PEG antibodies) (**Figure 1**).

Importantly, a recent study demonstrated that the vaccination against SARS-CoV-2 with mRNA-1273 (Moderna) and BNT162b2 (Pfizer-BioNTech) can lead to the *de novo* formation of anti-PEG antibodies as well to an increase of pre-existing anti-PEG antibodies (16, 17). Although anti-PEG antibodies had no impact on SARS-CoV-2-specific neutralizing antibody response to vaccination (17) *in vitro*, little is known about the impact of anti-PEG antibodies on other PEGylated drugs such as PRX-102. In this study, we performed a comprehensive work-up to analyze the frequencies and biochemical impact of anti-PEG and amino acid-specific anti-AGAL antibodies on PRX-102 in FD patients [n=102] naïve to PRX-102.

## 2 Methods

### 2.1 Blood samples

**Figure 2:**
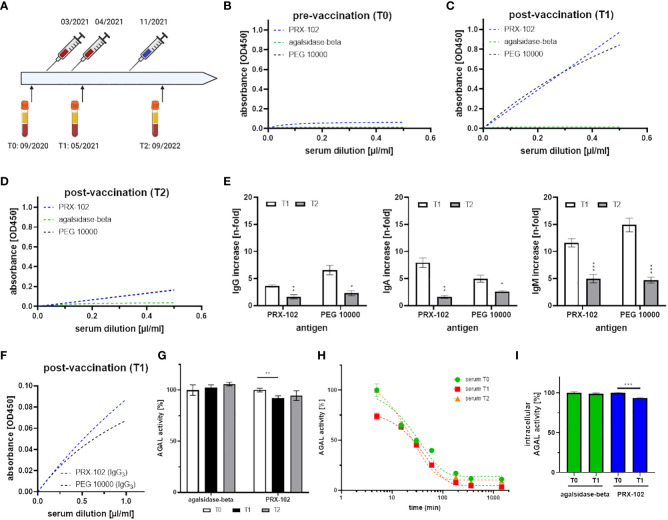
Detailed characterization of serum samples and antibody formation against PEG after mRNA-based SARS-CoV-2-vaccination. **(A)** Time-line for vaccination (basic immunization and 1st booster: mRNA-1273 [Moderna], 2nd immunization with BNT162b2 [Pfizer-BioNTech]) and blood drawing. **(B-D)** anti-IgG (total) detection in T0, T1 and T2 against PEG 10000 (black), PEGylated PRX-102 (blue), and the negative control agalsidase-beta (green). **(E)** Total IgG, IgA and IgM increase in T1 and T2 over time (compared to T0). **(F)** IgG-isotypization reveals signals for IgG3 against PEG 10000 (black) and PEGylated PRX-102 (blue). **(G)** Serum-mediated inhibition measurements against agalsidase-beta and PRX-102 with sera from T0, T1 and T2. **(H)** Enzymatic stability assays with PRX-102 in sera from T0 (green), T1 (red), and T2 (orange). **(I)** Cellular uptake analyses with agalsidase-beta (green) and PRX-102 (blue) with sera from T0 and T1. AGAL, α-galactosidase A; PEG, polyethylene glycol; PRX-102, pegunigalsidase-alfa. *p<0.05, **p<0.01, ***p<0.001.

All investigations were performed after approval by the Medical Association of Westphalian-Lippe and the Ethics Committee of the Medical Faculty of the University of Muenster (project no. 2011- 347-f, date of report: July 7, 2011) and in accordance with the Declaration of Helsinki. Written informed consent was obtained from the participant and patients for analysis and publication. The participant was a 41 year old healthy male. Basic immunization against SARS-CoV-2 and the first booster were performed with mRNA-1273 (Moderna) Spikevax vaccine, the second booster was performed with BNT162b2 (Pfizer-BioNTech) Comirnaty vaccine. Blood drawing was done before the basic vaccination (T0), one month after the first booster (T1) and 10 months after the second booster (T2) (**Figure 2**). FD patients were recruited consecutively at the Interdisciplinary Fabry Center Muenster (IFAZ) between 07/2021 and 10/2022. Blood sampling for antibody measurements was performed at least one week after infusions to minimize potential interferences with infused enzymes. For 14 patients with known vaccination status additional blood samples were retrospectively analyzed.

### 2.2 Enzyme-linked immunosorbent assays

Appropriate ELISAs to detect anti-PEG antibodies were performed as follows. Wells of 96-well plates were pre-coated with either 100 ng agalsidase-beta (Sanofi Genzyme), 100 ng pegunigalsidase-alfa (PRX-102; Chiesi GmbH) both solved in phosphate-buffered saline (PBS) or 20 µg polyethylene glycol (PEG-10.000; 81280, Sigma Aldrich, Hamburg, Germany) solved in 0.1 M NaHCO3/Na2CO3 (adjusted to pH 9.5 with HCl) buffer over night at 4°C. Of note, the α-galactosidase A enzyme agalsidase-beta is an approved drug for ERT in FD and served as a non-PEGylated AGAL control, sharing high amino acid similarity with PRX-102. (10, 11) Plates were subsequently washed three times with PBS, blocked with 2% (w/v) skim milk powder or bovine serum albumin (BSA) in PBS for one hour, and incubated overnight at 4°C with serial dilutions of raw sera. Wells were washed three times with 0.1% Chaps/PBS and two times with PBS. Detection antibodies were used as shown in **Supplementary Table 1** and incubated for 2 hours in 2% (w/v) skim milk powder or BSA in PBS. All detection antibodies except anti-IgG3 were HRP-coupled and did not require secondary amplification. Anti-IgG3 was detected using goat anti-rabbit IgG-HRP. The wells were again washed three times with 0.1% Chaps/PBS and two times with PBS. Finally, 50 µl 1-step TMB-ELISA substrate solution (Thermo Fisher Scientific, Darmstadt, Germany) was added to each well, followed by 50 µl 2 M sulfuric acid to stop the reaction after 15 to 30 min. Absorption was measured at 450 nm in a plate reader (M200 Infinite Pro, TECAN, Crailsheim, Germany). ELISAs were performed in duplets or triplets.

### 2.3 Serum-mediated inhibition assays

Serum-mediated inhibition assays were performed as previously described (6, 7). In short, 5 µl of patients’ sera were preincubated with 1 ng pegunigalsidase-alfa (PRX-102; Chiesi GmbH) or 1 ng agalsidase-beta (Sanofi Genzyme) for 10 min at room temperature. Subsequently, 4-methylumbelliferyl-β-D-galactopyranoside (Santa Cruz Biotechnology, Heidelberg, Germany) was added to measure α-galactosidase A (AGAL) activity via fluorescence measurement. N-acetylgalactosamine (Santa Cruz Biotechnology) was used to specifically block endogenous α-galactosidase B activity. After 1 h incubation at 37°C, fluorescence activity was measured at 460 nm. Five µl FCS, instead of human serum, were used as a control. Detected AGAL activity was expressed as a percentage compared to activity measured in the control. Measurements were performed in triplets. The inhibitory capacities of individual antibody titers was measured against agalsidase-alfa, agalsidase-beta and PRX-102 as previously described (8).

### 2.4 Enzymatic stability assays of PRX-102 and agalsidase-beta

To determine effects of anti-PEG antibodies on PRX-102 half-life in serum, 1 ng PRX-102 or agalsidase-beta were incubated with 10 µl serum over time (T0: 0 min, T1: 5 min, T2: 15 min, T3: 30 min, T4: 60 min, T5: 180 min, T6: 360 min, T7: 1440 min) at 37°C. Remaining AGAL activities were determined as described above. To express AGAL activity in percentage, AGAL activities from T0 were set as 100% in patients with no anti-AGAL antibodies. In patients with high inhibition, values were compared to controls (NaCl). Enzymatic stability assays were measured in triplets.

### 2.5 Enzyme uptake assays

Enzyme uptake assays were performed as recently described (13). In short, AGAL-deficient EA.hy926 cells were seeded on 96-well plates with a density of 2 x 10^5^ cells/ml and grown until confluence. To determine the effect of neutralizing anti-PEG antibodies on cellular AGAL uptake, 10 µl sera were pre-incubated with 5 µg/ml AGAL (agalsidase-beta or PRX-102, respectively) for 10 min at room temperature. Subsequently, mixtures were added to cells and incubated for 4 h at 37°C. Cells were then washed with PBS and for subsequent enzyme activity assays, cells were lysed with 30 µl 1x Passive Lyse Buffer (Promega, Wisconsin, USA, E194A). AGAL activities were determined as described above and normalized for protein concentrations.

### 2.6 Statistical analysis

Non-linear fitting models were used to demonstrate a serum dilution-dependent signal for ELISAs. Two-tailed Student’s t tests or one-way analyses of variance (ANOVAs) with Dunnett’s multiple comparison tests were used to compare effects of sera on antibody binding and AGAL activities. Statistical significance was considered at a two-sided p<0.05. If not stated otherwise data are shown as mean with standard deviation. Some figures were performed with BioRender. GraphPad Prism v.5.0 software (GraphPad Software, La Jolla, CA, USA) was used for appropriate statistical analyses and visualization.

## 3 Results

### 3.1 Impact of mRNA-mediated SARS-CoV-2 vaccination on anti-PEG antibody formation

Here, we demonstrate the *de novo* formation of anti-PEG antibodies in a healthy male after the second mRNA-based vaccination against SARS-CoV-2 (**Figure 2**). The male tolerated all vaccinations well and showed only mild typical SARS-CoV-2 vaccination-related symptoms. We identified the anti-PEG antibodies as IgG3, IgA and IgM isotypes (**Figures 2B–F**), pointing towards a thymus-independent immune reaction. Of note, no IgG1, IgG2, IgG4 and IgE antibodies were detected (data not shown). Strikingly, the anti-PEG antibodies recognized PRX-102, which has recently been approved as a new PEGylated enzyme replacement therapy for FD (12). Our subsequent functional characterization showed an inhibitory function of anti-PEG antibodies towards PRX-102 (**Figure 2G**), leading to a decreased serum half-life *in vitro*, resulting in a 2.15-fold decreased area under the curve (AUC, **Figure 2H**). Finally, the presence of anti-PEG antibodies led to a significantly reduced intracellular enzyme activity in cell culture (**Figure 2I**). Although antibody titers (IgA, IgG, IgM) decreased significantly over time (p<0.01), they were still detectable 10 months after the third vaccination (**Figure 2E**). The observed antibody titer reduction could be due to the longer interval to the last vaccination (2nd booster) or explained by the use of a different vaccine (BNT162b2), which seems to have less influence on anti-PEG formation (16).

### 3.2 Frequencies of anti-PEG antibodies in patients with Fabry disease

Since FD patients can be treated with PRX-102 in near future, it is most important to assess the presence of pre-existing anti-PEG antibodies in FD patients and analyze their potential impact on PRX-102 function (**Supplemental Figure 1**).

**Figure 3:**
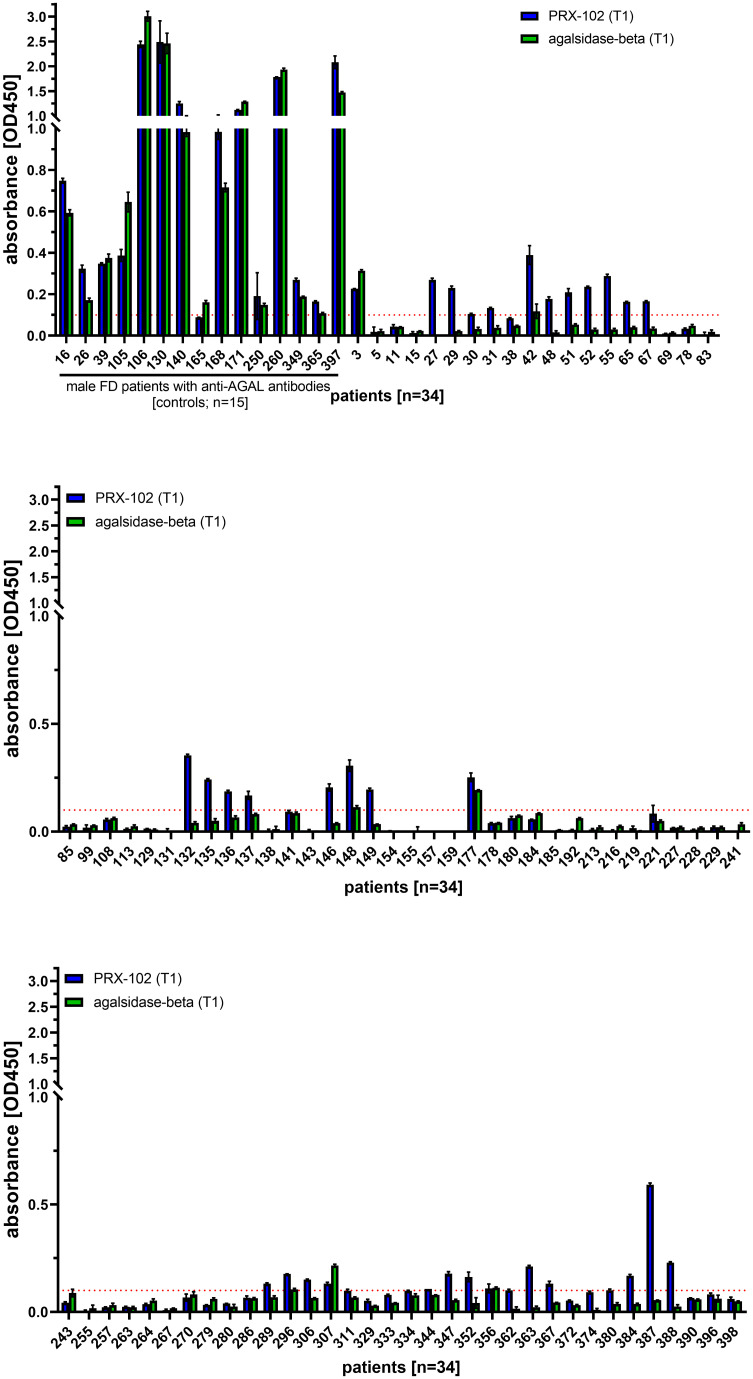
Evaluation of the ELISA-based antibody determination against pegunigalsidase-alfa and agalsidase-beta from 102 FD patients. ELISA against agalsidase-beta and pegunisgalsidase-alfa were performed in sera from 102 FD patients (males and females) including 15 male patients with known status of inhibitory anti-AGAL antibodies. The red dotted line marks the cut-off value for positive signals. PRX-102, pegunigalsidase-alfa.

To detect these antibodies, ELISAs against PRX-102 and agalsidase-beta (control) from 102 patients (46 [46.9%] males) were performed. At the time-point of blood drawing, 43 patients (29 [67.4%] males) received ERT (agalsidase-alfa or agalsidase-beta), 17 patients (10 [58.8%] males) received migalastat and 42 patients (7 [16.7%] males) were treatment-naïve. Of note, 15 (14.7%) male patients with neutralizing anti-AGAL antibodies (included as controls) were positive for an IgG response against PRX-102 and agalsidase-beta (due to cross-reactivity) (**Figure 3**). ELISAs against PRX-102 identified 29 out of 87 (33.3%) patients with an IgG response to PRX-102, whilst negative against agalsidase-beta (**Figure 3**). Of note, ELISA signals and thus titers from these patients were lower compared to titers from positive controls. In detail, ELISA-based signals from 15 patients with known status of inhibitory anti-AGAL antibodies compared to the other 87 patients, were 11.7-fold higher against PRX-102 and 17.1-fold higher against agalsidase-beta (both p<0.0001, respectively).

### 3.3 Impact of anti-PEG and anti-AGAL antibodies on enzymatic PRX-102 activities

Neutralizing anti-AGAL antibodies show a strong inhibitory effect against rhAGALs (6). Therefore, we analyzed the potential impact of pre-existing anti-PEG-specific antibodies on enzyme activities using standard serum-mediated inhibition assays and serum half-life assays. Of note, since high migalastat serum-concentrations interfere with AGAL activity assays, 17 patients receiving migalastat had to be excluded from further analyses and were grouped separately.

### 3.4 Serum-mediated inhibition assays

Individual data from serum-mediated inhibition assays against agalsidase-beta and PRX-102 are provided within the supplements (**Supplemental Figure 2**).

**Figure 4:**
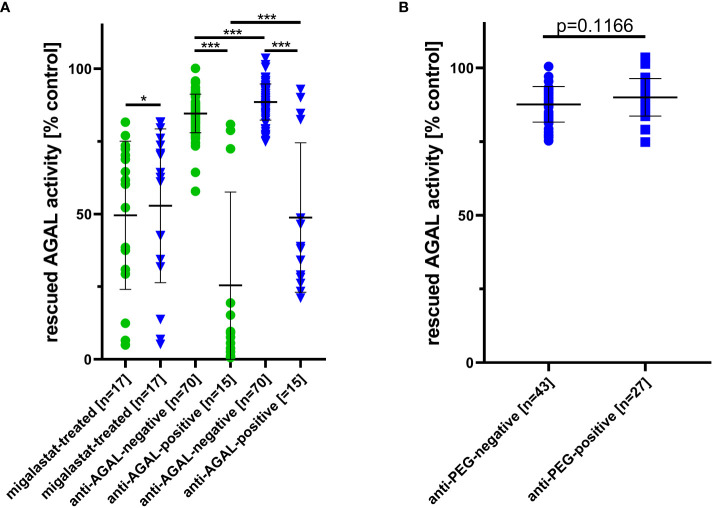
Outcomes for serum-mediated inhibition assays. **(A)** Impact of migalastat-treatment and inhibitory anti-AGAL antibody-negative and -positive sera against agalsidase-beta (circles) and PRX-102 (triangles). **(B)** Impact of anti-PEG antibodies in sera on PRX-102 activity. AGAL, α-galactosidase A; PRX-102, pegunigalsidase-alfa. *p<0.05, ***p<0.001.

Patients without anti-AGAL antibodies [n=70] showed a significantly higher rescued AGAL activity against agalsidase-beta [85 ± 7% versus 25 ± 32%; p<0.0001] and PRX-102 [89 ± 6% versus 49 ± 26%; p<0.0001] compared to those with anti-AGAL antibodies [n=15] (**Figure 4A**). In addition, sera from both anti-AGAL antibody-negative and -positive patients had significantly less inhibitory effect towards PRX-102 compared to agalsidase-beta (anti-AGAL antibody-negative: 89 ± 6% versus 85 ± 7%; p<0.0001; anti-AGAL antibody-positive: 49 ± 26% versus 25 ± 32%; p<0.0001] (**Figure 4A**), which confirms our recent data, demonstrating that pre-existing anti-AGAL antibodies show less affinity towards PRX-102 (13).

As noted above, sera from migalastat-treated patients showed a distinctive AGAL inhibition and thus reduced rescued AGAL activities in serum-mediated inhibition assays against agalsidase-beta (50 ± 26%) and PRX-102 (53 ± 26%). Of note, this inhibitory effect was slightly lower on PRX-102 (p=0.0129; **Figure 4A**).

To analyze an effect of anti-PEG antibodies on PRX-102 activities, we compared rescued AGAL activities between anti-PEG antibody-negative [n=43] and -positive patients [n=27]. Serum-mediated inhibition assays showed no differences for rescued AGAL activities between both groups [88 ± 6% versus 90 ± 6%; p=0.1555] (**Figure 4B**).

### 3.5 Serum half-life assays

**Figure 5:**
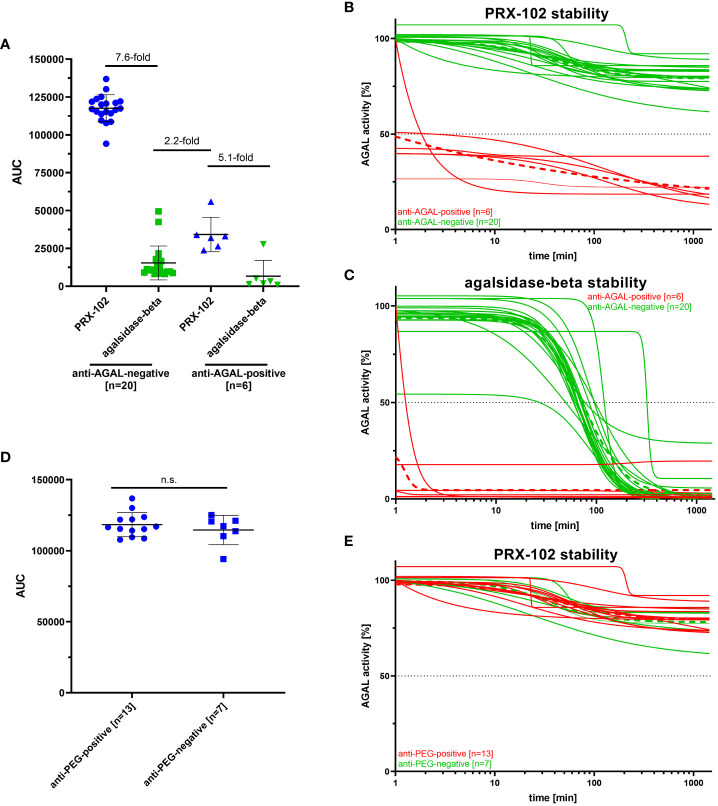
Outcomes for enzyme stability assays for anti-AGAL-negative and -positive and anti-PEG-negative and -positive patients. **(A)** Evaluation of serum stabilities of PRX-102 and agalsidase-beta in sera from anti-AGAL-negative and -positive patients. **(B)** Individual PRX-102 stability for anti-AGAL-positive and -negative sera. **(C)** Individual agalsidase-beta stability for anti-AGAL positive and -negative sera. **(D)** Evaluation of serum stabilities of PRX-102 in sera from anti-PEG-negative and -positive patients. **(E)** Individual PRX-102 stability for anti-PEG-positive and -negative sera. Green dotted lines represents mean values for anti-AGAL- and anti-PEG-negative patients. Red dotted lines represent mean values for anti-AGAL- and anti-PEG-positive patients. ADA, anti-drug antibody; AGAL, α-galactosidase A; AUC, area under the curve; n.s., not significant; PRX-102, pegunigalsidase-alfa.

PRX-102 shows an increased half-life compared to agalsidase-beta (10, 11). To determine the influence of anti-AGAL and anti-PEG antibodies on the serum half-life of both enzymes, we additionally performed stability assays with sera from a subset of patients (n=26), including anti-AGAL antibody-positive (n=6) and -negative (n=20) patients (**Figure 5**). AUC in anti-AGAL-negative patients (n=20) for PRX-102 was 7.6-fold higher compared to agalsidase-beta, reflecting the prolonged half-life of PRX-102 (**Figure 5**). As expected, AUCs of PRX-102 and agalsidase-beta in anti-AGAL-positive sera (n=6) was significantly decreased. However, the AUC of PRX-102 in anti-AGAL-positive sera was still approximately 33% compared to negative sera and thus 5-fold higher than for agalsidase-beta (**Figure 5A**).

Next, we analyzed whether pre-existing anti-PEG antibodies affect serum-half-life of PRX-102 in anti-AGAL antibody-negative patients (n=20) (**Figure 5**). The presence of anti-PEG antibodies in the sera from 13 patients had no significant effects on serum half-life of PRX-102 (**Figures 5D, E** **)**.

### 3.6 Impact of mRNA-mediated SARS-CoV-2 vaccination on anti-PEG antibody formation

Our initial data (**Figure 2**) showed an immune response, resulting in a *de novo* formation of anti-PEG antibodies with inhibitory function towards PRX-102 due to mRNA-mediated SARS-CoV-2 vaccination. Since FD patients will be treated with PRX-102 in the near future, we retrospectively analyzed whether mRNA-mediated SARS-CoV-2 vaccination might have also led to the formation of inhibitory anti-PEG antibodies in the general FD population. Thus, we additionally retrospectively analyzed sera of 14 patients with known vaccination status (vaccinated with mRNA-1273 [Moderna] or BNT162b2 [Pfizer-BioNTech]). The mean duration between vaccination and blood sampling was 7.5 ± 4.5 months. Enzyme stability assays with PRX-102 showed no significant differences between both visits (**Supplemental Figure 3**).

## 4 Discussion

Our main results are: 1) mRNA-mediated SARS-CoV-2 vaccination can lead to a *de novo* formation of anti-PEG antibodies, which can have an inhibitory function towards PRX-102 but decrease in the absence of further antigen presentation. 2) The presence of pre-existing anti-PEG antibodies was 33% in our FD patients. 3) The low titers of pre-existing anti-PEG antibodies seem to have little inhibitory effect on enzyme activity and stability *in vitro*. 4) Pre-existing anti-AGAL antibodies reduce serum half-life of PRX-102 *in vitro* significantly by ~60%. However, the resulting AUC is still 5-fold higher compared to agalsidase-beta.

PEGs comprise a family of hydrophilic polymers widely used in medical, pharmaceutical, cosmetic, industrial and processed food products. Exposure extends from the household to perioperative settings, and PEGs are common constituents of a variety of products including wound dressings, PEGylated drugs, laxatives and hydrogels (14). The high exposure to PEG results in an immune response eventually leading to immune globulins (Igs) against PEG in up to one third of the population (18). In addition, a recent study demonstrated the *de novo* formation and/or titer increase of anti-PEG antibodies due to mRNA-mediated SARS-CoV-2 vaccination (16). Although these antibodies had no neutralizing effect on vaccination efficacy *in vitro (* 16) their (inhibitory) impact on other PEGylated drugs was not analyzed.

Since PRX-102, a second-generation ERT for FD, is a PEGylated enzyme, it is important to assess and monitor the potential impact of anti-PEG antibodies on PRX-102 activity. To get an overview of the overall functional significance of ADAs, antibodies in FD need to be differentiated as anti-AGAL antibodies, which recognize the amino acid chain, and anti-PEG antibodies, which recognize PEG residues on PRX-102. In our cohort, ~1/3 of patients presented with pre-existing anti-PEG antibodies, which corresponds to the frequency of occurrence in the general population (up to 40%) before COVID-19 pandemia (18). While anti-AGAL antibodies generally show an inhibitory function, the effect of anti-PEG antibodies appears to be more variable and especially titer-dependent since only the serum of the healthy male showed marked inhibitory anti-PEG antibody-mediated inhibition shortly after vaccination. One reason that we did not observe an effect of vaccination on anti-PEG antibody titers in our FD patients could be the longer interval between vaccination and blood sampling and testing. Ju and colleagues (16) tested their patients after ~3 weeks post vaccination. While our initial control subject presented with increased anti-PEG titers 1 month after the first booster, blood samples from our FD patients were collected in average of 7.5 months after vaccination. In this setup, it is conceivable that anti-PEG antibody titers have already decreased, as shown for the control subject at 10 months after the 2^nd^ booster. To analyze this in more detail, appropriate studies should be designed with blood sampling and testing at 1 to 2 months after vaccination. Since the effect of anti-PEG antibodies depends on the titer, future studies are required to establish a cut-off value for specific anti-PEG antibody titers associated with measurable biochemical effects on AGAL activity and, more importantly, disease progression in affected patients treated with PRX-102.

In a previous study, we demonstrated that pre-existing anti-AGAL antibodies show less affinity against PRX-102 (13). These data were confirmed by our current study, demonstrating that serum half-life of PRX-102 was less severely impaired by pre-existing anti-AGAL antibodies compared to agalsidase-beta. Of note, the resulting half-life was still 2.2-fold higher compared to the serum half-life of agalsidase-beta in sera from patients without pre-existing anti-AGAL bodies. Future studies are now warranted to assess if PRX-102-treated patients with anti-AGAL antibodies will also benefit clinically, showing better disease outcome than patients with anti-AGAL antibodies receiving agalsidase-alfa or agalsidase-beta.

Our ELISA-based data demonstrated significant higher antibody titers against agalsidase-beta as well as PRX-102 in patients with known inhibitory anti-AGAL antibodies. However, since not all samples were measured on one plate and ADA titers could not be quantified due to the lack of a proper reference antibody against PEG, these data should be interpreted carefully and are yet preliminary. Nonetheless, again a difference of ~33% between anti-PRX-102 and anti-agalsidase-beta antibody titers confirms that pre-existing ADAs show less affinity against PRX-102.

Although the analysis of the impact of migalastat on AGAL activities in migalastat-treated patients was not a scope of this study, we claimed an interesting and important clinical result. Sera from migalastat-treated patients show a striking inhibitory capacity against agalsidase-beta and PRX-102. Thus, it can be assumed that AGAL activity measures, which are performed to document biochemical amenability of certain migalastat-treated patients will often result in false negative results due to the inhibitiory function of migalastat. This might explain why some studies have failed to demonstrate the expected increase of AGAL activity in migalastat-treated patients over time (19, 20). In this respect, future studies are required to assess whether migalastat-induced inhibitory capacity decreases between 2 consecutive time points of intake, which could identify the optimal time-point for measuring AGAL activity measures during follow-up. Regardless, serum-mediated inhibition assays appear to be a useful tool to monitor patient medication adherence to migalastat.

The frequency of pre-existing anti-PEG antibodies in patients with FD is comparable to that in the general population. Due to the low titers, these pre-existing antibodies had little effect on PRX-102 activity *in vitro*. However, treatment with PEGylated drugs such as mRNA-based COVID-19 vaccination might increase titers at least in short term. In this respect, we also like to highlight here that the identified anti-PEG antibodies are a side effect of the vaccination and may not be mixed up with the intended antibodies against SARS-CoV2, which have a longer half-life. So far, mainly male patients were positive for anti-AGAL antibodies (due to the absence of endogenous AGAL). Since female patients also develop anti-PEG antibodies, future antibody screenings in PRX-102-treated patients need to include female patients, too. Future studies including an isotyping of anti-PEG antibodies in FD patients treated with pegunigalsidase-alfa will be important. Furthermore, the effects of PEGylated drugs such as mRNA-based vaccinations on anti-PEG antibody formation need to be assessed in these patients. Due to the prolonged half-life of PRX-102, the development of a suitable test will be a challenge, as most antibody assays are based on the detection of free antibodies (13, 21). However, serum sampling for antibody measurements should be performed directly before the next infusion, which would ensure the lowest possible plasma PRX-102 concentration and highest free antibody concentration.

It is conceivable that therapy efficacy may be better under next-generation PRX-102 therapy than under current ERTs in terms of reduced inhibitory effects of anti-AGAL and minor (marginal or insignificant) inhibitory effect of anti-PEG antibodies. Since neutralizing antibodies may reduce the efficacy of ERT treatment in FD patients, patients on PRX-102 therapy should be tested for anti-PEG and anti-AGAL antibodies before and during treatment.

## 5 Limitations

Only PRX-102-naïve patients (approved 5^th^ May 2023; availability of the drug for treatment expected in October 2023) were included in this study, thus no effects of PRX-102 treatment on antibody formation could be analyzed, which will be the scope of a future study. We did not differentiate between male and female patients, since both sexes are cross-reactive immunological material (CRIM)-negative for PEG and thus will probably have a comparable risk to form anti-PEG antibodies. We focused on IgG determination in FD patient’s sera, because these Igs were the most abundant in our initial healthy subject after vaccination. Therefore, the presence of IgM antibodies in addition to IgG antibodies cannot be excluded. The use of 10k PEG instead of 2k PEG might be a limitation. However, we were able to identify antibodies recognizing the PEG residue on PRX-102, which was at least the goal of this study. Furthermore, we did not perform enzyme uptake assays with patients’ sera to analyze enzyme uptake, activity and Gb_3_ reduction intracellularly, since sample material was limited and we focused on the identification and biochemical characterization of these antibodies. However, we demonstrate that high anti-PEG titers can influence the enzyme uptake, which was also demonstrated for anti-AGAL antibodies, previously (13). Future studies are now required to assess a potential cellular PEG accumulation, as well as antigen-antibody-complexes especially in patients treated with pegunigalsidase-alfa. The unknown vaccination status in most recruited FD patients is a limitation. Future studies including PRX-102-treated patients are now warranted to address these limitations.

## Data availability statement

The original contributions presented in the study are included in the article/**Supplementary Material**. Further inquiries can be directed to the corresponding author.

## Ethics statement

The studies involving humans were approved by Medical Association of Westphalian-Lippe and the Ethics Committee of the Medical Faculty of the University of Muenster. The studies were conducted in accordance with the local legislation and institutional requirements. The participants provided their written informed consent to participate in this study.

## Author contributions

ML: Conceptualization, Formal Analysis, Funding acquisition, Investigation, Methodology, Project administration, Resources, Supervision, Visualization, Writing – original draft. LF: Formal Analysis, Investigation, Methodology, Writing – original draft. S-MB: Resources, Writing – review & editing. EB: Conceptualization, Funding acquisition, Resources, Supervision, Writing – review & editing.
