# Supplementary material for: Characterization of pre-existing anti-PEG and anti-AGAL antibodies towards PRX-102 in patients with Fabry disease
Source: Front Immunol. 2023 Sep 22;14:1266082. doi: 10.3389/fimmu.2023.1266082 (PMC10561604; doi:10.3389/fimmu.2023.1266082)
Supplement: Supplementary file 1 [file DataSheet_1.docx]

Supplementary Material

| **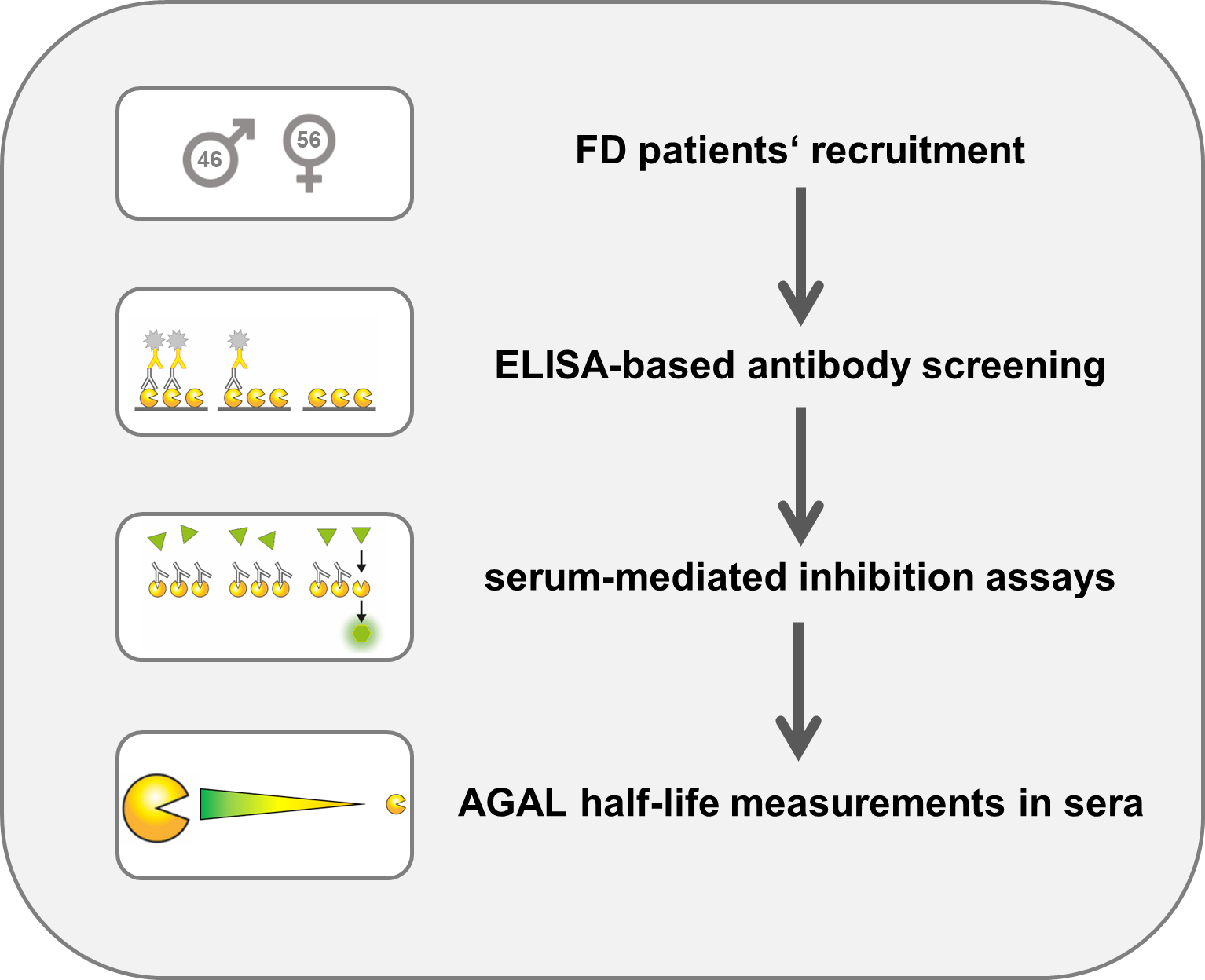** |
| --- |
| **Supplemental Figure 1: Overview of the study.** In total, 56 female and 46 male Fabry disease (FD) patients were consecutively recruited and screened for anti-PEG and anti-AGAL antibodies. AGAL inhibition of individual sera was measured by serum-mediated inhibition assays. In a subset of patients’ sera, effects of anti-AGAL and anti-PEG antibodies in patients’ sera on agalsidase-beta and pegunigalsidase-alfa was measured. AGAL: α-galactosidase A, PEG: polyethylene glycol. |

|  |
| --- |
| **Supplemental Figure 2: Individual serum-mediated inhibition measures.** -: anti-AGAL-inhibition-negative; +: anti-AGAL-inhibition-positive; m: migalastat-treated. AGAL: α-galactosidase A. PRX-102: pegunigalsidase-alfa. |
|  |
| **Supplemental Figure 3: Comparison of the half-life of PRX-102 in human sera before and after mRNA-mediated SARS-CoV-2 vaccination (n=14).** AUC: area under the curve. |

| **Supplemental Table 1: Antibodies used for ELISAs.** | | | | |
| --- | --- | --- | --- | --- |
| target | article number | company | host species | working dilution |
| α-human total-IgG | ab98624 | Abcam | goat | 1:15.000 |
| α-human IgA | ab97215 | Abcam | goat | 1:10.000 |
| α-human IgE | ab99806 | Abcam | mouse | 1:10.000 |
| α-human IgM | ab97205 | Abcam | goat | 1:10.000 |
| α-human IgG1 | ab99774 | Abcam | mouse | 1:2.000 |
| α-human IgG2 | ab99779 | Abcam | mouse | 1:2.000 |
| α-human IgG3 | ab86253 | Abcam | rabbit | 1:2.000 |
| α-human IgG4 | ab99823 | Abcam | mouse | 1:2.000 |
| α-rabbit IgG | 12-348 | Merck | goat | 1:5.000 |
